# Upregulation of Long Non-Coding RNA GCC2-AS1 Facilitates Malignant Phenotypes and Correlated With Unfavorable Prognosis for Lung Adenocarcinoma

**DOI:** 10.3389/fonc.2020.628608

**Published:** 2021-01-26

**Authors:** Fengqiang Yu, Mingqiang Liang, Weidong Wu, Yu Huang, Jiantao Zheng, Bin Zheng, Chun Chen

**Affiliations:** ^1^ Department of Thoracic Surgery, Fujian Medical University Union Hospital, Fuzhou, China; ^2^ Fujian Provincial Key Laboratory of Cardiothoracic Surgery, Fujian Medical University Union Hospital, Fuzhou, China

**Keywords:** lncRNA, GCC2-AS1, lung adenocarcinoma, The Cancer Genome Atlas, prognosis

## Abstract

**Background:**

The role played by long noncoding RNA GCC2-AS1 in primary malignant tumors remains poorly understood. This study aimed to determine the expression levels and evaluate the clinical significance and biological effects of GCC2-AS1 in lung adenocarcinoma (LUAD).

**Methods:**

We used data obtained from tissue samples and the TCGA database to determine the levels of GCC2-AS1 expression LUAD patients, and the prognostic value of those levels. Functional experiments were performed to investigate the effect of GCC2-AS1 on LUAD cells. Genes that were differentially expressed in GCC2-AS1-low and -high groups were analyzed by an enrichment analysis. Additionally, a nomogram model was created and subgroup analyses were performed to further determine the prognostic value of GCC2-AS1 in LUAD.

**Results:**

GCC2-AS1 expression was significantly upregulated in lung adenocarcinoma tissues as compared with normal tissues. Depletion of GCC2-AS1 inhibited the proliferation and invasion of LUAD cells *in vitro*. An elevated level of GCC2-AS1 was strongly correlated with shorter overall survival time and was identified as an independent prognostic marker for LUAD patients. Enrichment analyses conducted using GO, KEGG, and GSEA databases were performed to identify biological pathways that might involve GCC2-AS1. Several subgroups were found to have a significant prognostic value for patients in the GCC2-AS1-low and -high groups.

**Conclusions:**

Our findings suggest that GCC2-AS1 can serve as a diagnostic and prognostic biomarker for LUAD patients.

## Introduction

Lung cancer is the leading cause of cancer-related morbidity and mortality worldwide (1). Non-small cell lung cancer (NSCLC) is the most common type of lung cancer, and accounts for 85% of all human lung cancers. As a major subtype of NSCLC, lung adenocarcinoma (LUAD) accounts for ~ 40% of all lung cancers, and is usually diagnosed at an advanced stage (2). While revolutionary breakthroughs have been achieved in lung cancer diagnosis and treatment, including the use of low-dose computed tomography and the discovery of molecular targeted therapeutic drugs, the 5-year survival rate for lung cancer patients is only 17.4% (3). Although several diagnostic or prognostic biomarkers have been identified for LUAD, such as carbohydrate antigen 125 (CA125), cytokeratin 19 fragment 21-1 (CYFRA21-1), and carcinoembryonic antigen (CEA), their actual clinical utility needs further investigation (4). Thus, there is an urgent need to identify reliable biomarkers for use in diagnosis and predicting the clinical outcomes of LUAD patients to improve their overall time (OS) time.

LncRNAs comprise a class of long non-coding RNA molecules that are >200 nucleotides in length, and are now regarded as novel transcripts that regulate the initiation and development of various solid malignant tumors (5, 6). Accumulating evidence suggests that aberrantly expressed lncRNAs are linking to the recurrence and prognosis of lung cancer (7–9). The long non-coding RNA GCC2-AS1 (GRIP and coiled-coil domain-containing protein 2 antisense RNA 1) is located on chromosome 2, and has an orientation opposite of the GCC2 gene. Recently, Jiang identified a rare fusion product of ALK (Anaplastic Lymphoma Kinase, GCC2-ALK) in a patient with advanced LUAD (10). This is similar to EML4-ALK, which is the most common ALK fusion found in patients with NSCLC. Dysregulation of GCC2-ALK can activate ALK downstream signaling (11), suggesting that GCC2 exerts a critical influence on the development of LUAD. Previous studies demonstrated that an antisense transcript can alter the expression levels of a sense gene by forming sense-antisense pairs (12, 13). However, the role played by GCC2-AS1 in LUAD remains poorly understood.

In the present study, we detected and compared the levels of GCC2-AS1 in LUAD tissues and normal tissues, as well as in LUAD cell lines and a normal lung epithelial cell line. We also detected the location and biological effects of GCC2-AS1 in the cell lines. Furthermore, human LUAD data from The Cancer Genome Atlas (TCGA) were downloaded and analyzed to explore the prognostic value of GCC2-AS1 and the relationship between GCC2-AS1 expression and various clinical parameters in LUAD patients. A gene set enrichment analysis (GSEA) was performed to gain a more in-depth understanding of GCC2-AS1-related biological pathways involved in LUAD. Our results shed light on the crucial role played by GCC2-AS1, and suggest GCC2-AS1 as a possible prognostic biomarker for LUAD.

## Materials and Methods

### Patient Samples

Samples of lung adenocarcinoma tissue (n = 75) and normal lung tissue (n = 50) were obtained from patients at the Fujian Medical University Union Hospital between September 2018 and December 2019. All samples were confirmed by pathological diagnosis and none of the donor patients had received any other type of treatment prior to surgery. The study protocol was approved by the Ethics Committee of Fujian Medical University Union Hospital, and all patients provided their written informed consent.

### Cell Culture and Transfection

Four human LUAD cell lines (H292, H1975, H1299, and A549) and a normal lung epithelial cell line (BEAS-2B) were acquired from the Cell Bank of the Chinese Academy of Sciences. All the cell lines were cultured in RPMI-1640 medium (Gibco, Waltham, MA, USA) containing 10% fetal bovine serum (FBS, Gibco) and 1% penicillin-streptomycin (HyClone, Logan, UT, USA) in a humidified 37°C incubator with a 5% CO_2_ atmosphere.

Small interfering RNA sequences against GCC2-AS1 (si-GCC2-AS1) and a negative control (NC) sequence were obtained from GenePharma (Shanghai, China). For siRNA transfection, cells were cultured and then transfected with si-GCC2-AS1 or the NC using Lipofectamine 3000 (Invitrogen, Carlsbad, CA, USA) as described in the manufacturer’s instructions.

### RNA Extraction and Reverse Transcriptase Quantitative Real-Time PCR (RT-qPCR)

Total RNA was extracted using TRIzol reagent (Invitrogen) and then reverse transcribed to cDNA using a PrimeScript™ RT reagent kit (Takara, Shiga, Japan) according to manufacturer’s instructions. GCC2-AS1 expression was determined by using SYBR Green GoTaq Master Mix (Promega, Madison, WI, USA) on an ABI 7500 thermocycler (Applied Biosystems Foster City, CA, USA). The specific primers used for GCC2-AS1 (Forward: ACCTGGTCTGGATCGGTCAC; Reverse: GGGCATGTTCTTCTTGACTGC) were synthesized by GenePharma (Shanghai, China). An 18S ribosomal RNA was used as internal control, and 2 ^-ΔΔ Ct^ method was utilized to calculate the relative expression of GCC2-AS1 (14).

### RNA-Fluorescence *In Situ* Hybridization (FISH)

Digoxin (DIG)-labeled GCC2-AS1, a modified probe for FISH, was synthesized by Servicebio (Wuhan, China). The sequence of the probe was 5’-DIG-CCGCCATCCTTGTTGTAGGGAACTCG-DIG-3’. A549 and H1299 cells were seeded into a confocal dish, and the subsequent procedures were performed as previously described (15). Images were captured with a confocal microscope (Nikon, Japan).

### Cell Counting Kit-8 (CCK-8) Assay

Cells were seeded into the wells of 96-well plates (3,000 cells/well) containing 200 μl of RPMI-1640 medium. The medium was refreshed every 24 h. Next, CCK8 solution (10 μl) was added to each well, and the cells were incubated for 2 h. Finally, the absorbance of each well at a wavelength of 450 nm was measured with a microplate reader.

### Transwell Assay

Cell invasion assays were performed using a 24-well Transwell chamber (Corning, NY, USA) coated with Matrigel (Corning). Cells (1.5 × 10^5^) suspended in 1% FBS medium were added to the upper chamber and 600 μl of 10% FBS medium was added to the lower chamber. After 24 h of incubation, cells that had invaded the lower chamber were fixed with 4% paraformaldehyde (PFA) and stained with 1% crystal violet.

### RNA-Sequencing Data and Bioinformatics Analysis

RNA-seq data and corresponding clinical information obtained from various LUAD projects (513 patients, Workflow Type: HTSeq-FPKM) were downloaded from the TCGA database and subjected to further analysis. Later, level 3 HTSeq-FPKM data was transformed into TPM (transcripts per million reads) data for subsequent analysis. Some cases with unavailable or unknown clinical characteristics were considered as missing values. Data containing a differential expression analysis of GTEx (Genotype-Tissue Expression) or a pan-cancer analysis were retrieved from University of California Santa Cruz (UCSC) XENA (https://xenabrowser.net/datapages/). RNAseq data for TPM obtained from the TCGA and GTEx analysis were continuously processed using Toil software (16). Based on GCC2-AS1 expression, the median expression value was used as a cut-off value, and tumor samples were then categorized into low and high groups, respectively. This study was conducted in accordance with publication guidelines provided by TCGA (https://cancergenome.nih,gov/publications/publicationguidelines).

### Identification of Differentially Expressed Genes (DEGs)

The DESeq2 R package was used to obtain expression profiles (HTSeq-Counts) that were subsequently used to make comparisons between the low and high GCC2-AS1 expression groups. A log (fold change) ||log (FC)|>1.5 and an adjusted *P*-value<0.05 were considered to constitute a threshold value for DEGs. Volcano plots and a heat map of DEGs were created using the gglot2 package (version 3.1.0, https://gihub.com/tidyverse/ggplot2) and pheatmap package (version 1.0.10, https://CRAN.R-project.org/package=pheatmap), respectively.

### Enrichment Analysis

The clusterProfiler package (17) was used to perform a gene ontology (GO) enrichment analysis of the input gene list, which included Biological Process (BP), Cellular Components (CC), and Molecular Function (MF). The same package was used to perform a KEGG pathway enrichment analysis. Furthermore, a gene set enrichment analysis (GSEA) was also performed using the clusterProfiler R package to determine the critical functions and pathways that involved the previously defined gene sets in the low and high GCC2-AS1 expression groups. Gene set permutations were carried out 1,000 times for each analysis. Based on |Normalized Enrichment Score||NES|>1, P-value <0.05, and false discovery rate (FDR) q value <0.25, a notably enriched pathway was identified for each phenotype.

### Statistical Analysis

All experimental data were analyzed using IBM SPSS Statistics for Windows, Version 22.0 software (IBM Corporation, Armonk, NY, USA). Statistical analyses of bioinformatics data were performed using R (v.3.5.1). Wilcoxon rank sum tests were used to compare the levels of GCC2-AS1 expression between lung adenocarcinoma and normal groups. Pearson’s chi-square test was used to investigate the relationship between GCC2-AS1 expression and clinicopathologic parameters. Additionally, a univariate Cox regression analysis and the Kaplan-Meier method were used to analyze the association between clinicopathologic parameters and OS time. We also conducted multivariate Cox regression analyses to identify independent prognostic factors. The individual hazard ratio (HR) and 95% confidence interval (95% CI) were determined to assess the hazard risk of each parameter. Subsequently, valuable clinicopathological parameters identified from multivariate Cox regression analyses were used to construct a nomogram for predicting the prognosis of LUAD patients. The nomogram was constructed using the rms package in R (Version: 5.1-3; https://cran.r-project.org/web/packages/rms/index.html). Additionally, calibration curves were created to check the nomogram’s predictive efficiency. A ROC (receiver operating characteristic) analysis was carried out using the pROC package (18) in R to estimate the model’s ability to discriminate between lung adenocarcinoma and normal groups. All statistical tests were 2-sided and a *P*-value <0.05 was considered to be statistically significant.

## Results

### Expression Pattern of GCC2-AS1 in LUAD as Determined in TCGA

**Figure 1:**
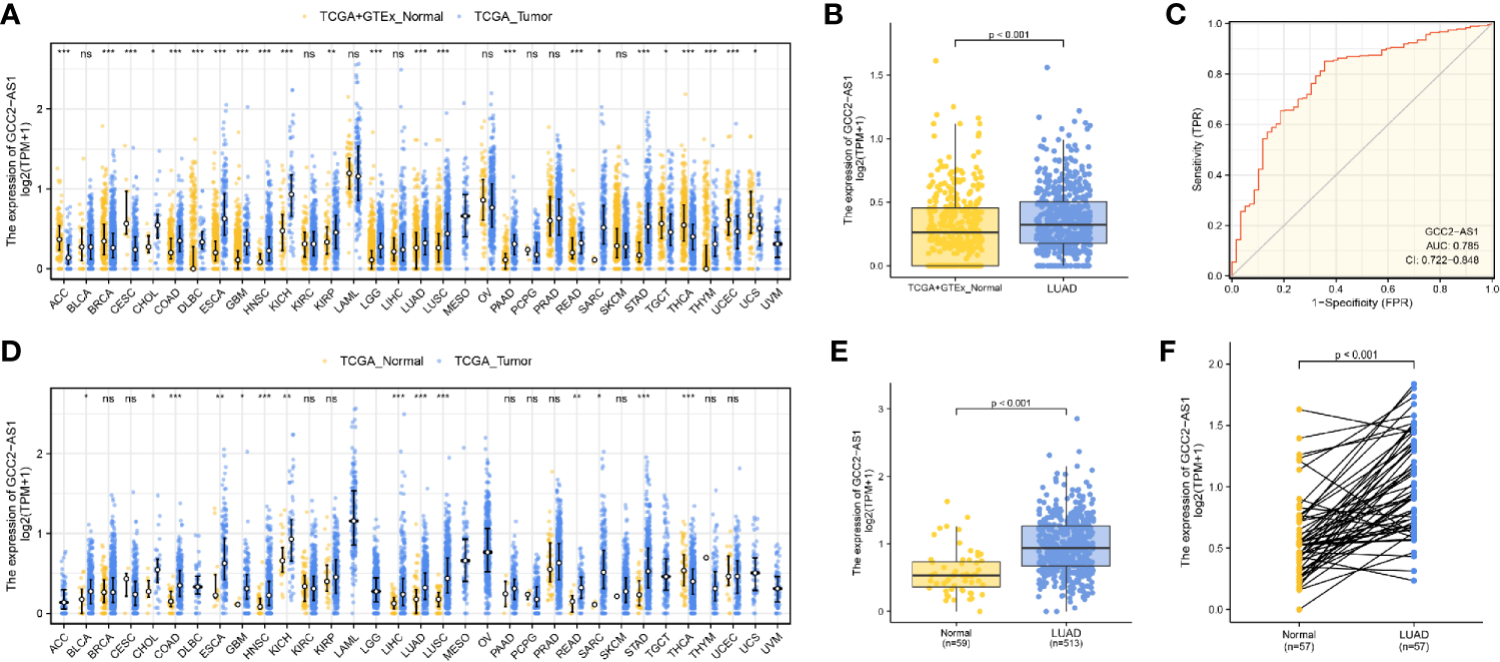
Expression patterns of GGC2-AS1 in primary malignant tumors as recorded in the TCGA database. Expression of GCC2-AS1 in all TCGA primary tumor samples **(A)** and LUAD samples **(B)** when compared with normal tissue samples. **(C)** ROC analysis of GCC2-AS1 expression showing its high ability to discriminate tumor from non-tumor samples. Expression of GCC2-AS1 in all TCGA primary tumor samples **(D)** and LUAD samples **(E)** when compared with the corresponding adjacent tissue samples. **(F)** Expression of GCC2-AS1 in LUAD samples and adjacent paired samples. ns, not significant; *P < 0.05; **P < 0.01; ***P < 0.001.

We initially downloaded RNA-seq data from the UCSC XENA databases and then used Toil workflow (16) software to transform it into TPM data for the purpose of detecting the patterns of GCC2-AS1 expression in 33 pan-cancer and normal tissue sample by performing Wilcoxon rank sum tests. As shown in **Figures 1A** and **1D**, levels of GCC2-AS1 expression were compared with normal tissues and adjacent tissues in online database, respectively. Surprisingly, significantly higher levels of GCC2-AS1 expression were found in the LUAD tissues when compared with the normal tissues (**Figure 1B**). An ROC analysis showed that GCC2-AS1 had a high ability to discriminate between LUAD patients and healthy individuals, with an AUC of 0.785 (**Figure 1C**), suggesting that GCC2-AS1 could be used to help identify people with LUAD. Furthermore, we demonstrated that GCC2-AS1 expression was upregulated in the LUAD samples (n = 513) when compared with its expression in samples of adjacent tissue (n = 59, **Figure 1E**). We next used the Wilcoxon signed rank test to compare the levels of GCC2-AS1 expression in 57 samples of LUAD tissue with those in paired adjacent tumors. The results showed that GCC2-AS1 was overexpressed in the LUAD samples (**Figure 1F**). These data suggested that GCC2-AS1 might function as an oncogene that facilitates LUAD carcinogenesis.

### Expression of GCC2-AS1 in Lung Adenocarcinoma and Its Distribution

**Figure 2:**
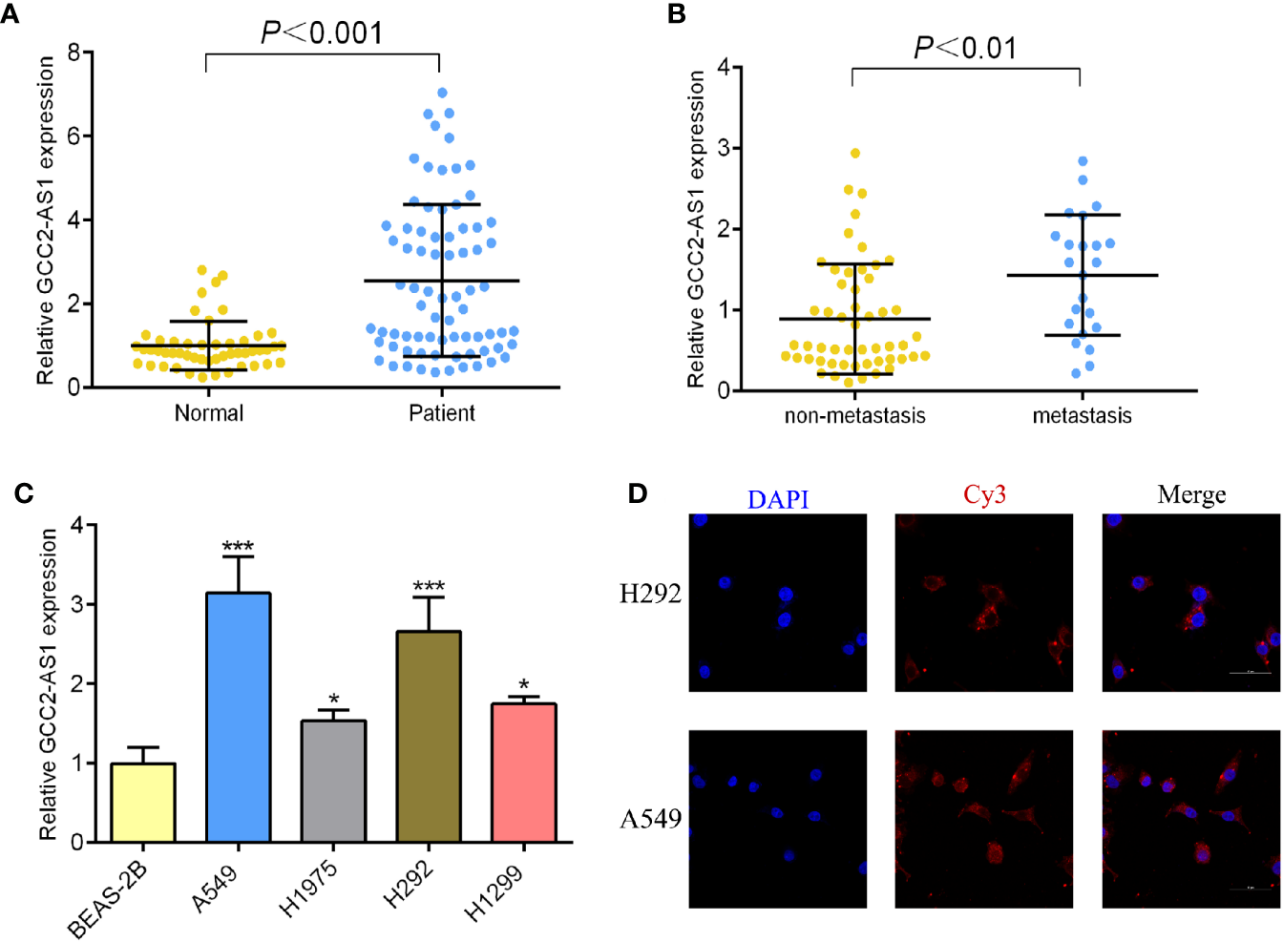
Expression of GCC2-AS1 in our study. **(A)** Expression of GCC2-AS1 in LUAD tissues and normal tissues. **(B)** Expression of GCC2-AS1 in metastatic tissues and non-metastatic tissues. **(C)** Expression of GCC2-AS1 in LUAD cell lines (H292, H1975, H1299, and A549) and a normal lung epithelial cell line, BEAS-2B. **(D)** FISH analysis showing the location distribution of GCC2-AS1 in LUAD cell lines. *P < 0.05; ***P < 0.001.

To determine whether GCC2-AS1 was differentially expressed in LUAD tissues, we collected 75 samples of LUAD tissue and 50 samples of normal lung tissue and analyzed them by RT-qPCR. We found that the levels of GCC2 expression in the LUAD tissues were extremely upregulated when compared those in normal lung tissues (**Figure 2A**) which is consistent with online database we discovered. We also found that GCC2-AS1 was more highly expressed in the LUAD tissues obtained from metastatic patients (n = 23) than from non-metastatic patients (n = 52, **Figure 2B**). Moreover, a similar result was found in a comparison of the LUAD cell lines with the normal lung epithelial cell line (**Figure 2C**). Finally, we found that GCC2-AS1 was mainly distributed in the cytoplasm of both A549 and H292 cells (**Figure 2D**).

### Association Between GCC2-AS1 Expression and Clinical Characteristics

The clinical characteristics of 513 LUAD patients (276 females and 237 males, mean age = 63 years) and their levels of GCC2-AS1 expression were obtained from the TCGA cohort (**Table 1**) We then divided those patients into two groups (a high and low expression group GCC2-AS1, respectively) based on the median value for GCC2-AS1 expression. An analysis of the total cohort showed that the parameters of age (*P* = 0.151), T stage (*P* = 0.111), N stage (*P* = 0.577), M stage (*P* = 0.095), pathologic stage (*P* = 0.084), primary therapy outcome (*P* = 0.962), race (*P* = 0.267), residual tumor (*P* = 0.721), anatomic neoplasm subdivision (Left/Right, *P* = 0.272; central/peripheral, *P* = 0.755), and tumor status (*P* = 0.311) were not associated with GCC2-AS1 expression. In contrast, a correlation analysis conducted using the chi square test showed that a high level of GCC2-AS1 expression was significantly correlated with gender (*P* = 0.007), smoker (*P* = 0.012), Tumor Protein P53 (TP53) mutation status (*P*<0.001), and Kirsten Rat Sarcoma Viral Oncogene (KRAS) mutation status (*P* = 0.011). Another analysis conducted using a similar method revealed that a high level of GCC2-AS1 expression was significantly associated with N stage (*P* = 0.031) and TNM stage (*P* = 0.005) (**Table 2**).

**Table 1:** Association between expression of GCC2-AS1 and clinical characteristics of LUAD patients from TCGA database.

Characteristics	Level	Low expression of GCC2-AS1	High expression of GCC2-AS1	*p* value
n		257	256	
Gender (%)	Female	154 (59.9%)	122 (47.7%)	**0.007**
	Male	103 (40.1%)	134 (52.3%)	
Age (%)	≤65	111 (44.8%)	127 (51.6%)	0.151
	>65	137 (55.2%)	119 (48.4%)	
T stage (%)	T1	95 (37.1%)	73 (28.7%)	0.111
	T2	133 (52.0%)	143 (56.3%)	
	T3	22 (8.6%)	25 (9.8%)	
	T4	6 (2.3%)	13 (5.1%)	
N stage (%)	N0	172 (68.5%)	158 (63.2%)	0.577^a^
	N1	42 (16.7%)	53 (21.2%)	
	N2	36 (14.3%)	38 (15.2%)	
	N3	1 (0.4%)	1 (0.4%)	
M stage (%)	M0	177 (95.7%)	167 (90.8%)	0.095
	M1	8 (4.3%)	17 (9.2%)	
Pathologic stage (%)	Stage I	148 (58.7%)	126 (49.8%)	0.084^a^
	Stage II	55 (21.8%)	66 (26.1%)	
	Stage III	41 (16.3%)	43 (17.0%)	
	Stage IV	8 (3.2%)	18 (7.1%)	
Primary therapy outcome (%)	CR	168 (74.7%)	147 (73.1%)	0.962
	PD	34 (15.1%)	34 (16.9%)	
	PR	3 (1.3%)	3 (1.5%)	
	SD	20 (8.9%)	17 (8.5%)	
Race (%)	Asian	5 (2.2%)	2 (0.9%)	0.267^a^
	Black or African American	22 (9.8%)	30 (13.6%)	
	White	198 (88.0%)	189 (85.5%)	
Residual tumor (%)	R0	171 (96.1%)	173 (94.5%)	0.721^a^
	R1	6 (3.4%)	7 (3.8%)	
	R2	1 (0.6%)	3 (1.6%)	
Anatomic neoplasm subdivision (%)	Left	93 (37.3%)	106 (42.6%)	0.272
	Right	156 (62.7%)	143 (57.4%)	
Anatomic neoplasm subdivision2 (%)	Central Lung	33 (34.4%)	29 (31.2%)	0.755
	Peripheral Lung	63 (65.6%)	64 (68.8%)	
Smoker (%)	No	47 (19.1%)	27 (10.7%)	**0.012**
	Yes	199 (80.9%)	226 (89.3%)	
Tumor status (%)	Tumor free	151 (65.4%)	137 (60.4%)	0.311
	With tumor	80 (34.6%)	90 (39.6%)	
TP53 status (%)	Mut	91 (35.7%)	150 (59.3%)	**<0.001**
	WT	164 (64.3%)	103 (40.7%)	
KRAS status (%)	Mut	83 (32.5%)	56 (22.1%)	**0.011**
	WT	172 (67.5%)	197 (77.9%)	

Significant results (p ＜ 0.05) are given in bold.

LUAD, lung adenocarcinoma; CR, complete response; PD, progressive disease; PR, partial response; SD, stable disease; Mut, mutant type; WT, wild type.

^a^Fisher’s exact test.

**Table 2:** Association between expression of GCC2-AS1 and clinical characteristics of LUAD patients from our database.

Characteristics	Level	Low expression of GCC2-AS1	High expression of GCC2-AS1	*p* value
n		37	38	
Gender (%)	Female	19 (51.3%)	14 (36.8%)	0.206
	Male	18 (48.7%)	24 (63.2%)	
Age (%)	≤65	26 (70.3%)	19 (50.0%)	0.073
	>65	11 (29.7%)	19 (50.0%)	
Smoker (%)	No	25 (67.6%)	26 (68.4%)	0.937
	Yes	12 (32.4%)	12 (31.6%)	
Tumor size (%)	≤3cm	27 (73.0%)	21 (55.3%)	0.11
	>3cm	10 (27.0%)	17 (44.7%)	
N stage (%)	N0	30 (81.1%)	22 (57.9%)	**0.031**
	N1	3 (8.1%)	2 (5.3%)	
	N2	4 (10.8%)	14 (36.8%)	
TNM stage (%)	Stage I	27 (73.0%)	14 (36.9%)	**0.005**
	Stage II	6 (16.2%)	10 (26.2%)	
	Stage III	4 (10.8%)	14 (36.9%)	

Significant results (p ＜ 0.05) are given in bold.

LUAD, lung adenocarcinoma.

### Knockdown of GCC2-AS1 Inhibited the Proliferation and Metastasis of LUAD Cells

**Figure 3:**
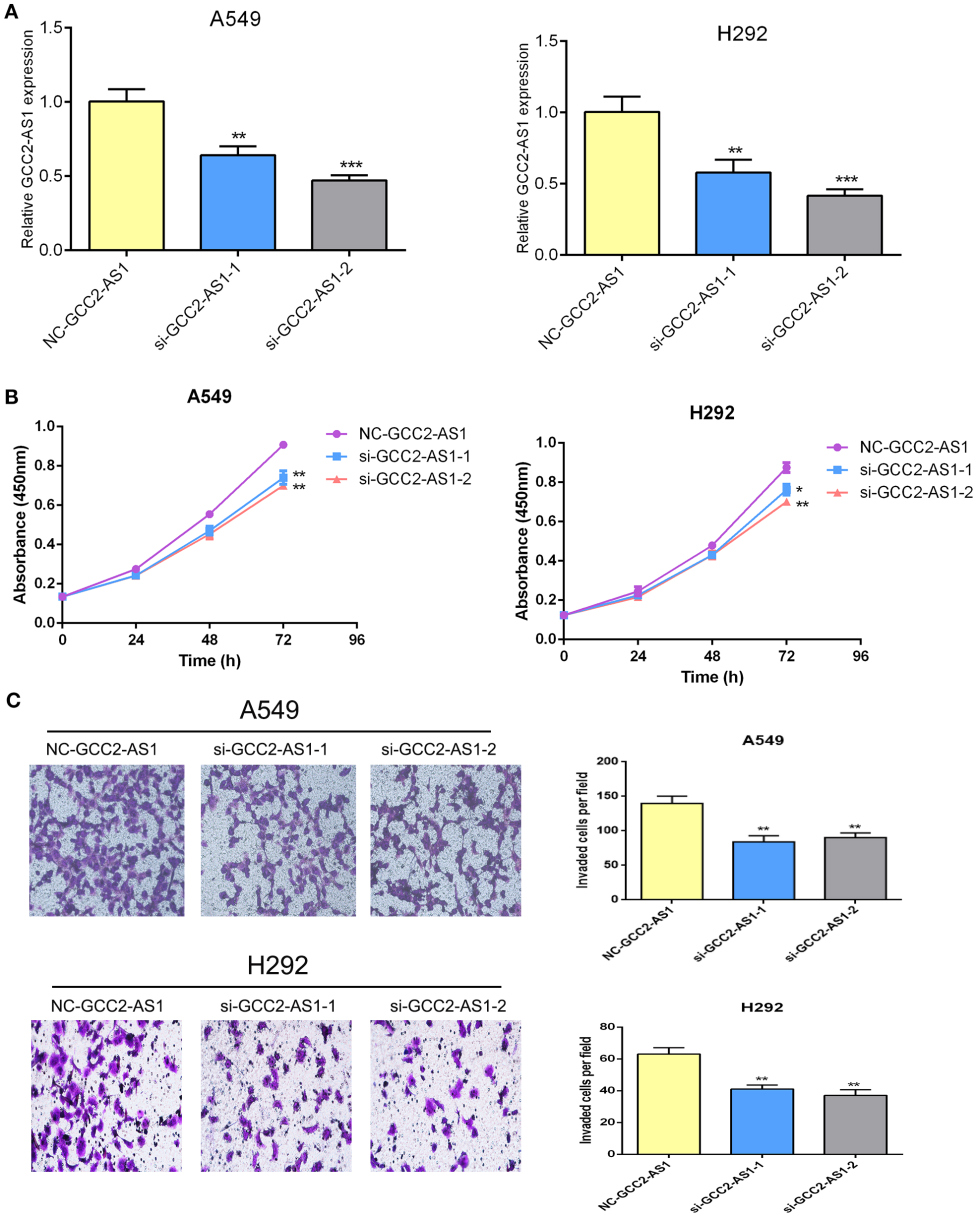
Effects of GCC2-AS1 on lung adenocarcinoma cells. **(A)** Relative expression of GCC2-AS1 in A549 and H292 cells transfected with GCC2-AS1 siRNA and negative control. **(B)** CCK8 assays was performed to detected the proliferation rate. **(C)** Transwell assays were assessed the invasive ability of each group. Scale bar, 20 μm. Data are presented as the mean ± SD of three independent experiments (**P < 0.01; ***P < 0.001).

To explore the biological effects of GCC2-AS1 on LUAD cells, we decreased the GCC2-AS1 levels in LUAD cells by transient transfection with siRNA. Transfection efficiency was determined by RT-qPCR after 48 h (**Figure 3A**). CCK-8 assays were conducted to detect the influence of GCC2-AS1 on LUAD cell proliferation. The results suggested that decreased GCC2-AS1 levels significantly suppressed the proliferation of LUAD cells when compared with cells in the NC groups (**Figure 3B**). Furthermore, Transwell assays were performed to examine how GCC2-AS1 affected the metastatic ability of LUAD cells. Depletion of GCC2-AS1 levels in the A549 and H292 cell lines notably reduced the numbers of invaded cells (**Figure 3C**). These data showed that GCC2-AS1 could enhance the proliferation and invasion of LUAD cells.

### Identification of Differentially Expressed Genes

**Figure 4:**
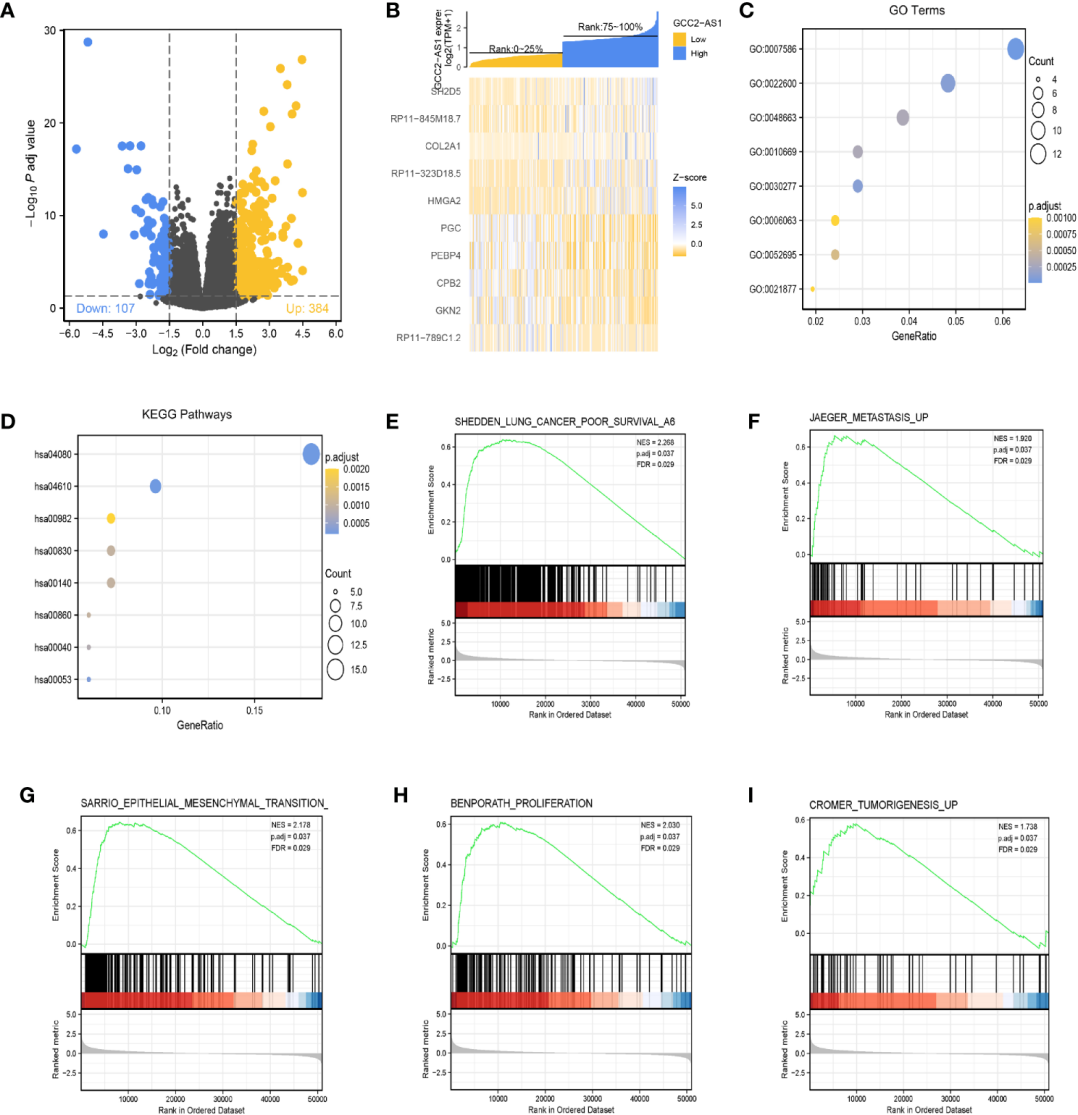
Identification of differentially expressed genes (DEGs) in the GCC2-AS1-low and -high patients and enrichment analysis in the TCGA database. **(A)** Volcano plot of DEGs. **(B)** Heat map of the top 10 DEGs. **(C)** GO enriched terms colored by *P*-value. **(D)** KEGG enriched terms colored by *P*-value. GSEA results revealing that poor survival **(E)**, metastasis **(F)**, epithelial mesenchymal transition **(G)**, proliferation **(H)**, and tumorigenesis **(I)** were significantly were enriched in the high GCC2-AS1 expression group. NES, normalized ES; FDR, false discovery rate.

The “DESeq2” R package was used to perform DEGs analyses of LUAD samples from the high and low GCC2-AS1 expression groups as identified in the TCGA database. Based the threshold value (|log(FC)|>1.5 and adjusted *P*-value <0.05), a total of 361 genes (384 upregulated and 107 downregulated) were identified as being differentially expressed (**Figure 4A**). The top 10 genes with positive and negative correlations in the low and high GCC2-AS1 expression groups are shown in **Figure 4B**.

### KEGG and GO Enrichment Analysis of DEGs

The R package clusterProfiler was used to performed a GO enrichment analysis of GCC2-AS1-related DEGs involved in several BPs, MFs, and CCs. The results (**Figure 4C**, **Supplementary Table 1**) showed that the genes for BPs (81 terms) were mainly involved with digestion, cellular glucuronidation, and regulation of respiratory gaseous exchange. The genes for MFs (three terms) and CC (one term) were mainly involved with glucuronosyltransferase activity, extracellular matrix structural constituent, DNA-binding transcription activator activity, RNA polymerase II-specific, and Golgi lumen, respectively. Similarity, a KEGG (13 terms, **Supplementary Table 2**) enrichment analysis revealed that the DEGs were predominately related to chemical carcinogenesis, gluconeogenesis, and the peroxisome proliferators-activated receptors (PPAR) signaling pathway (**Figure 4D**, **Supplementary Table 2**).

### Gene Set Enrichment Analysis

Next, the R package clusterProfiler was used to perform a GSEA hallmark analysis to identify genes with critical involvement in signaling pathways in the low and high GCC2-AS1 expression groups of LUAD patients. All the enriched key pathways were identified according to significant differences (|NES|>1, P-value <0.05, and FDR q value <0.25) in the enrichment of genes in the MSigDB collection (C2.all.v6.2.symbols), including lung cancer poor survival (**Figure 4E**), metastasis (**Figure 4F**), epithelial mesenchymal transition (EMT, **Figure 4G**), proliferation (**Figure 4H**), and tumorigenesis (**Figure 4I**) in the high GCC2-AS1 expression phenotype, suggesting that GCC2-AS1 might play a role in LUAD progression.

### Prognostic Value and a Multivariate Analysis

**Figure 5:**
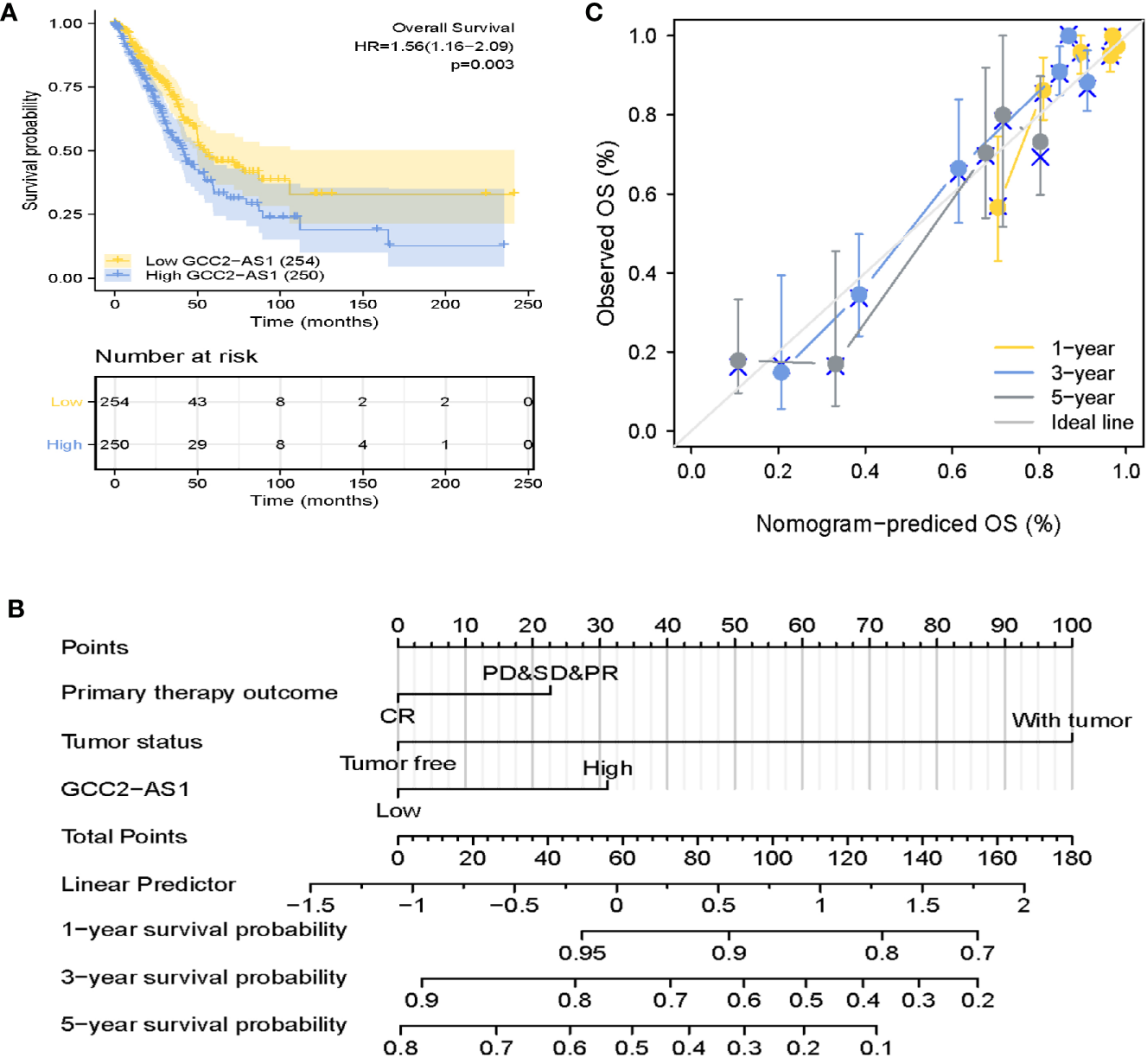
Prognostic role of GCC2-AS2 and a nomogram. **(A)** Kaplan-Meier OS curves for patients with low and high levels of GCC2-AS1 expression as extracted from the TCGA database. **(B)** Nomogram for predicting the probability of 1-, 3-, and 5-year OS for LUAD patients. **(C)** Calibration curve of the nomogram.

The survmier R package was used to perform a Kaplan-Meier analysis that evaluated the prognostic value of GCC2-AS1 in the GCC2-AS1-high and GCC2-AS1-low groups. The results showed that patients with high levels of GCC2-AS1 expression had shorter OS time than patients in the GCC2-AS1-low groups (**Figure 5A**). Univariate and multivariate Cox regression analyses were performed to further investigate factors that correlated with a patient’s prognosis (**Table 3**). The univariate analysis showed that T stage (HR = 1.668 [1.184–2.349], *P* = 0.003), N stage (HR = 2.606 [1.939–3.503], *P*<0.001), M stage (HR = 2.111 [1.232–3.616], *P* = 0.007), pathologic stage (HR = 2.975 [2.188–4.045], *P*<0.001), primary therapy outcome (HR = 2.818 [2.004–3.963], *P*<0.001), residual tumor (HR = 3.973 [2.217–7.120], *P*<0.001), tumor status (HR = 6.211 [4.258–9.059], *P*<0.001), and GCC2-AS1 expression (HR = 1.560 [1.164–2.092], *P* = 0.003) were significantly correlated with the OS time of LUAD patients (**Table 2**). A multivariate analysis revealed that GCC2-AS1 expression (HR = 1.816 [1.113–2.964], *P* = 0.017), primary therapy outcome [(HR = 2.442 [1.401–4.254], *P* = 0.002), and tumor status (HR = 6.028 [3.339–10.884], *P*<0.001) independently predicted overall time. These data suggest that GCC2-AS1 could serve as an independent prognostic factor for LUAD patients.

**Table 3:** Univariate and multivariate analysis of prognostic factors in LUAD patients for overall survival.

Characteristics	Univariate analysis		Multivariate analysis	
	*P* value	HR (95% CI)	*P* value	Hazard Ratio
T stage (T2 & T3 & T4 *vs.* T1)	**0.003**	1.668 (1.184–2.349)	0.151	1.571 (0.848–2.912)
N stage (N1 & N2 & N3 *vs.* N0)	**<0.001**	2.606 (1.939–3.503)	0.209	1.670 (0.750–3.718)
M stage (M1 *vs.* M0)	**0.007**	2.111 (1.232–3.616)	0.823	1.119 (0.419–2.984)
Pathologic stage (Stage II & Stage III & Stage IV *vs.* Stage I)	**<0.001**	2.975 (2.188–4.045)	0.58	0.782 (0.328–1.868)
Primary therapy outcome (PD & SD & PR *vs.* CR)	**<0.001**	2.818 (2.004–3.963)	**0.002**	2.442 (1.401–4.254)
Residual tumor (R1 & R2 *vs.* R0)	**<0.001**	3.973 (2.217–7.120)	0.19	1.910 (0.726–5.022)
Gender (Male *vs.* Female)	0.694	1.060 (0.792–1.418)		
Age (>65 *vs.* <=65)	0.171	1.228 (0.915–1.649)		
Race (White *vs.* Asian & Black or African American)	0.162	1.422 (0.869–2.327)		
Anatomic neoplasm subdivision (Right *vs.* Left)	0.878	1.024 (0.758–1.383)		
Anatomic neoplasm subdivision (Peripheral Lung *vs.* Central Lung)	0.706	0.913 (0.570–1.463)		
Number pack years smoked (>=40 *vs.* <40)	0.84	1.038 (0.723–1.490)		
Smoker (Yes *vs.* No)	0.568	0.887 (0.587–1.339)		
Tumor status (With tumor *vs.* Tumor free)	**<0.001**	6.211 (4.258–9.059)	**<0.001**	6.028 (3.339–10.884)
TP53 status (Mut *vs.* WT)	0.13	1.254 (0.936–1.680)		
KRAS status (Mut *vs.* WT)	0.623	1.087 (0.779–1.517)		
GCC2-AS1 (High *vs.* Low)	**0.003**	1.560 (1.164–2.092)	**0.017**	1.816 (1.113–2.964)

Significant results (p ＜ 0.05) are given in bold.

ESCC, lung adenocarcinoma; HR, hazard ratio; CI, confidence interval.

Furthermore, a nomogram that integrated GCC2-AS1 with other independent prognostic factors identified by the multivariate Cox analysis was created to evaluate the probability of accurately predicting a prognosis. As shown in **Figure 5B**, variables that contributed to the total score for each case were used to determine the probability of a LUAD patient’s survival at 1, 3, and 5 years. The C-index for the nomogram was 0.758 (95% CI: 0.733–0.782). The calibration curve was very close to the actual curve (45-degree line, **Figure 5C**), indicating strong agreement between the predicted probability and actual probability. In short, these findings indicated that the nomogram could provide a relatively accurate prediction of LUAD patient’s survival time.

### Prognostic Role of GCC2-AS1 in Subgroup Analyses

**Figure 6:**
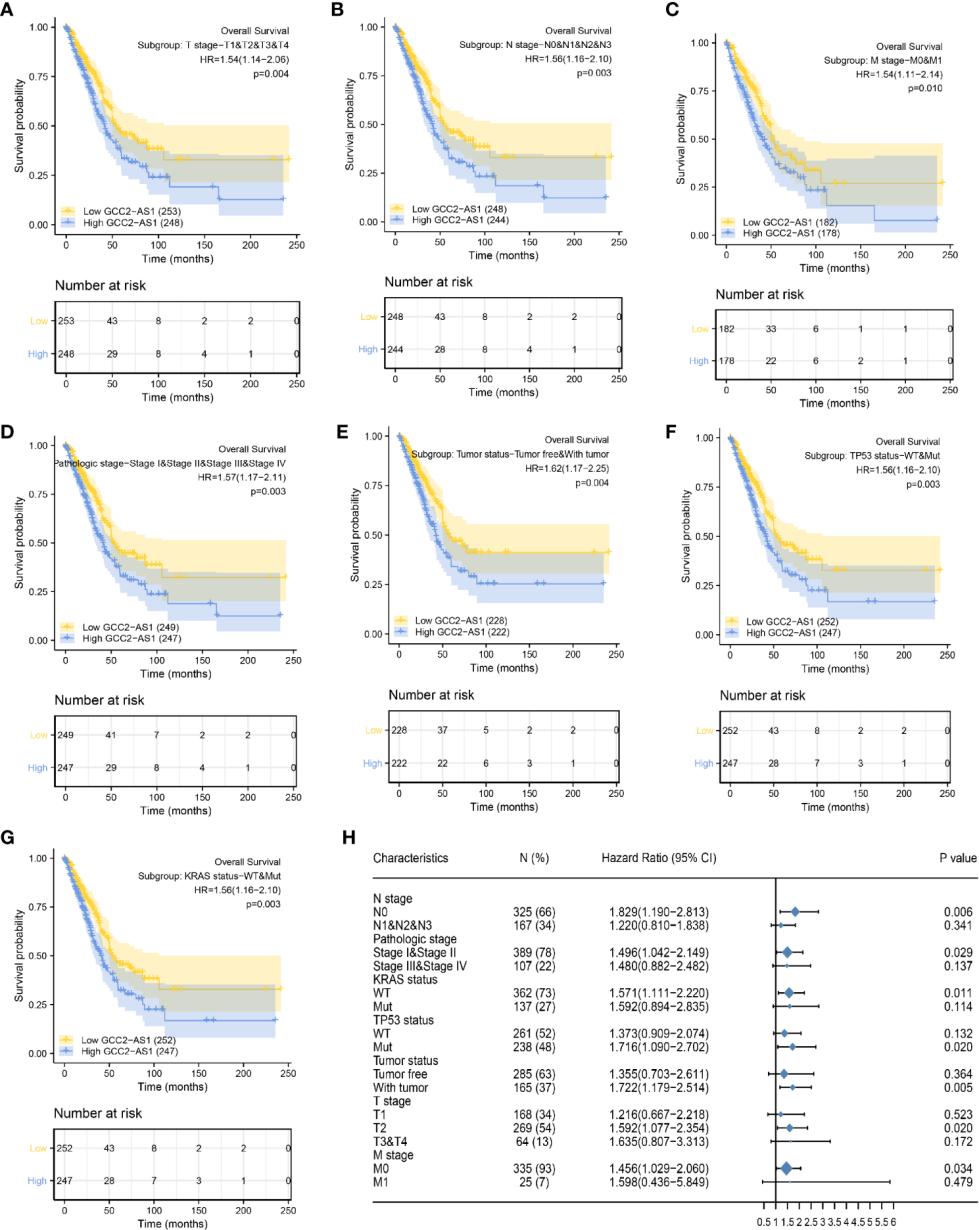
Kaplan Meier analysis of subgroups and creation of the forest plot. The GCC2-AS1-high groups had shorter OS time when compared with the GCC2-AS1-low groups in T stage **(A)**, N stage **(B)**, M stage **(C)**, pathological stage **(D)**, tumor status **(E)**, TP53 status **(F)**, and KRAS status **(G)**. **(H)** Forest plot showing the prognostic significance of GCC2-AS1 in various subgroups.

To better understand the factors that affect the prognosis of LUAD patients, we conducted stratification analyses by creating survival charts and a forest plot. These analyses showed that a high level of GCC2-AS1 expression was strongly correlated with shorter OS time among patients with T stage (**Figure 6A**), N stage (**Figure 6B**), M stage (**Figure 6C**), pathological stage (**Figure 6D**), tumor status (**Figure 6E**), TP53 status (**Figure 6F**), and KRAS status (**Figure 6G**). In addition, the forest plot based on the cox regression analysis showed that GCC2-AS1 expression levels were of prognostic significance in the N0 subgroup (HR = 1.829 [1.190–2.813], *P* = 0.006) of N stage, stage I & stage II subgroups (HR = 1.496 [1.042–2.149], *P* = 0.029) of pathological stage, WT group (HR = 1.571 [1.111–2.220], *P* = 0.011) of KRAS status, Mut subgroup (HR = 1.716 [1.090–2.702], *P* = 0.020) of TP53 status, tumor subgroup (HR = 1.722 [1.179–2.514], *P* = 0.005) of tumor status, T2 subgroup (HR = 1.592 [1.077–2.354], *P* = 0.020) of T stage, and M0 subgroup (HR = 1.456 [1.029–2.060], *P* = 0.034) of M stage (**Figure 6H**).

## Discussion

Lung cancer has ranked first in both morbidity and mortality among all cancers for many years, and remains a huge threat to public health (19). Despite recent discoveries of numerous genetic mutations associated with lung cancer and the development of immunotherapeutic agents and drugs, most LUAD patients initially present with advanced disease and have a poor overall 5-year survival rate of ~5% (3). Overwhelming evidence indicates that dysregulated lncRNAs participate in lung cancer initiation and progression (20–23), suggesting that those lncRNAs could serve as novel biomarkers for diagnosis and predicting the prognosis of lung cancer patients. The gene for long non-coding RNA GCC2-AS1 is located on chromosome 2q12.3 and might serve as an antisense transcript regulator of the GCC2 gene, which is thought to facilitate oligomerization of the ALK kinase domain, thereby leading to the constitutive activation of ALK kinase. GCC2-ALK, a novel targetable fusion product, was reported to be sensitive to ALK inhibitors used for patients with NSCLC (10, 24). This suggests that GCC2 could be a driver gene. No published study has specifically reported the levels of GCC2-AS1 expression in cancer, and this is the first study to determine the expression patterns and biological effects of GCC2-AS1 in LUAD.

Large-scale investigations that gather deep sequencing data are now widely conducted to characterize various cancers by analyzing their levels and types of lncRNA expression (20). We initially obtained RNA-seq data related to LUAD from TCGA and performed an in-depth analysis of the data. GCC2-AS1 has drawn our attention among dysregulated lncRNAs from results that it was remarkably upregulated in LUAD tissues when compared with normal or paired adjacent tissues. A high level of GCC2-AS1 expression in LUAD tissues was found to be associated with gender, smoker, TP53 mutation status, and KRAS mutation status. ROC analysis revealed that GCC2-AS1 has potential ability to discriminate patients from healthy people.

To validate the bioinformatic results, we next obtain surgically resected samples. Surprisingly, GGC2-AS1 was significantly overexpressed in LUAD patients compared with that in normal controls. Further analysis showed that GCC2-AS1 was highly expressed in LUAD tissues from patients with metastatic disease. Increased levels of GCC2-AS1 were significantly correlated with tumor size and TNM stage, which was largely different from our findings. This disparity might due to the limited number of samples in our study as well as racial differences. However, a similar trend was universally found in various types of malignant tumors, and the trend indicated that GGC2-AS1 might act as an oncogenic factor that mediates tumor initiation and progression. In addition, we also found obviously elevated levels of GCC2-AS1 expression in LUAD cell lines when compared with those levels in the BEAS-2B cell line. Of note, biological experiments showed that GCC2-AS1 could facilitate the proliferation and invasion of LUAD cells. A FISH analysis showed that GCC2-AS1 is predominantly located in the cytoplasm of both A549 and H292 cell lines. This finding suggests that it probably forms complexes with RNA-binding proteins (RBPs) or partially complementary mRNAs to regulate the stability and/or translation of specific mRNAs (25).

Abnormally regulated lncRNAs can modulate a gene signaling network at transcriptional, post-transcriptional, and post-translational levels (26). To gain a preliminary understanding of how GCC2-AS1 functions, genes that were differentially expressed in the high and low GCC2-AS1 expression groups were identified based on a bioinformatics analysis. Several significant terms in the GO and KEGG enrichment analysis, such as cellular glucuronidation, DNA-binding transcription activator activity, and PPAR signaling pathway were related to functions that could facilitate carcinogenesis (27). Furthermore, GSEA results revealed that high a level of GCC2-AS1 expression was strongly associated with poor survival, metastasis, EMT, proliferation, and tumorigenesis of lung cancer. Moreover, our *in vitro* experiments verified that GCC2-AS1 enhanced the proliferative and metastatic abilities of LUAD cells, and all the enrichment data sets indicated that GCC2-AS1 was strongly correlated with tumor initiation and development. However, additional experiments must be conducted to confirm the specific biological role of GCC2-AS1 in LUAD.

Prognosis of cancer patients is the key concern for doctors. We analyzed the prognostic value of GCC2-AS1, and observed that a high level of GCC2-AS1 expression was related to shorter OS time. A multivariate Cox regression analysis clearly showed that GCC2-AS1 expression, primary therapy outcome and tumor status are independent factors that predict the OS time of LUAD patients. The nomogram model a useful and prognostic tool to evaluate the personalized survival time (28), comprising independent factors had a relatively accurate predictive capability with a C-index of 0.758. Nevertheless, GCC2-AS1 did not account for a massive percentage of total points when compared with tumor status. This might be due to the limited sample size in our study and individual differences among patients. Further studies with much larger sample sizes and longer follow-up time are needed to confirm the accuracy of the nomogram model. To this end, we conducted a comprehensive Kaplan-Meier analysis and created a forest plot of a set of subgroups that showed a lower OS rate in the GCC2-AS1-high groups *versus* the GCC2-AS1-low groups, including T stage, N stage, M stage, pathological stage, tumor status, TP53 status, and KRAS status. The forest plot further demonstrated the prognostic significance of GCC2-AS1 in various subgroups, including the N0 subgroup of N stage, stage I & stage II subgroups of pathological stage, WT group of KRAS status, Mut subgroup of TP53 status, with the tumor subgroup of tumor status, T2 subgroup of T stage, and the M0 subgroup of M stage. This information provides guidelines for utilizing GCC2-AS1 levels to predict the OS times of LUAD patients. It is worth noting that TP53 and KRAS status are universally recognized as critical biomarkers for predicting a prognosis in LUAD, indicating that GCC2-AS1 integrated with TP53 and KRAS status might potentially improve surveillance OS time. These findings suggest that GCC2-AS1 could be utilized as both a diagnostic and prognostic biomarker for LUAD patients. Future investigations should explore the role of GCC2-AS1 in other types of malignant tumors.

This study has some limitations that should be mentioned. First, our data does not clearly indicate the prognostic value of GCC2-AS1 because of a lack of sufficient follow-up time. Second, this study included the size of samples are limited, further large-scale of samples needed to verify the trend would be convincing. Third, potential mechanism by which GCC2-AS1 contributes to LUAD carcinogenesis have not been adequately investigated. We will further explore the role of GCC-AS1 in LUAD to address the above questions.

Collectively, this study provides multi-level evidence that demonstrates the important role played by GCC2-AS1 in LUAD. We observed that an increased level of GCC2-AS1 expression was significantly correlated with a shorter OS time in LUAD patients. Our multivariate analysis showed that GCC2-AS1 could serve as an independent diagnostic and prognostic factor. The present study partially illustrates the role played by GCC2-AS1 in LUAD, and suggests its use as novel diagnostic and prognostic biomarker for LUAD patients.

## Data Availability Statement

The raw data supporting the conclusions of this article will be made available by the authors, without undue reservation.

## Ethics Statement

The studies involving human participants were reviewed and approved by the ethics committee of Fujian Medical University Union Hospital. The patients/participants provided their written informed consent to participate in this study.

## Author Contributions

CC conceived and designed the study. FY performed the experiment, analyzed data, and wrote the manuscript. ML and WW collected patients’ clinical data and analyzed the TCGA data. YH and JZ assisted with experimental performance and data analysis. BZ helped to revise the manuscript. All authors contributed to the article and approved the submitted version.

## Funding

This research was supported by Science and Technology Major Project of Fujian province (2017YZ0001) Medical Innovation project of Fujian Health and Family Planning Commission (2015-CX-19) and Provincial Science and Technology Leading Project (2018Y0032).

## Conflict of Interest

The authors declare that the research was conducted in the absence of any commercial or financial relationships that could be construed as a potential conflict of interest.

## References

[1] Siegel R Miller K Jemal A. Cancer statistics, 2019. CA Cancer J Clin (2019) 69(1):7–34. doi: 10.3322/caac.21551, PMID: 30620402

[2] Zappa C Mousa S. Non-small cell lung cancer: current treatment and future advances. Trans Lung Cancer Res (2016) 5(3):288–300. doi: 10.21037/tlcr.2016.06.07, PMID: 27413711 PMC4931124

[3] Siegel R Miller K Jemal A. Cancer statistics, 2016. CA Cancer J Clin (2016) 66(1):7–30. doi: 10.3322/caac.21332, PMID: 26742998

[4] Nakamura H Nishimura T. History, molecular features, and clinical importance of conventional serum biomarkers in lung cancer. Surg Today (2017) 47(9):1037–59. doi: 10.1007/s00595-017-1477-y, PMID: 28229299

[5] Jiang M Ni J Cui W Wang B Zhuo W. Emerging roles of lncRNA in cancer and therapeutic opportunities. Am J Cancer Res (2019) 9(7):1354–66., PMID: 31392074 PMC6682721

[6] Xiu B Chi Y Liu L Chi W Zhang Q Chen J. LINC02273 drives breast cancer metastasis by epigenetically increasing AGR2 transcription. Mol Cancer (2019) 18(1):187. doi: 10.1186/s12943-019-1115-y, PMID: 31856843 PMC6921600

[7] Loewen G Jayawickramarajah J Zhuo Y Shan B. Functions of lncRNA HOTAIR in lung cancer. J Hematol Oncol (2014) 7:90. doi: 10.1186/s13045-014-0090-4, PMID: 25491133 PMC4266198

[8] Su M Xiao Y Tang J Wu J Ma J Tian B. Role of lncRNA and EZH2 Interaction/Regulatory Network in Lung Cancer. J Cancer (2018) 9(22):4156–65. doi: 10.7150/jca.27098, PMID: 30519315 PMC6277609

[9] Qian H Chen L Huang J Wang X Ma S Cui F. The lncRNA MIR4435-2HG promotes lung cancer progression by activating β-catenin signalling. J Mol Med (Berl) (2018) 96(8):753–64. doi: 10.1007/s00109-018-1654-5, PMID: 29872866

[10] Jiang J Wu X Tong X Wei W Chen A Wang X. GCC2-ALK as a targetable fusion in lung adenocarcinoma and its enduring clinical responses to ALK inhibitors. Lung Cancer (Amsterdam Netherlands) (2018) 115:5–11. doi: 10.1016/j.lungcan.2017.10.011, PMID: 29290262

[11] El-Osta H Shackelford R. Personalized treatment options for ALK-positive metastatic non-small-cell lung cancer: potential role for Ceritinib. Pharmgenomics Pers Med (2015) 8:145–54. doi: 10.2147/pgpm.s71100, PMID: 26622190 PMC4638315

[12] Sun J Wang X Fu C Wang X Zou J Hua H. Long noncoding RNA FGFR3-AS1 promotes osteosarcoma growth through regulating its natural antisense transcript FGFR3. Mol Biol Rep (2016) 43(5):427–36. doi: 10.1007/s11033-016-3975-1, PMID: 27022737

[13] Kim W Miguel-Rojas C Wang J Townsend J Trail F. Developmental Dynamics of Long Noncoding RNA Expression during Sexual Fruiting Body Formation in Fusarium graminearum. mBio (2018) 9:e01292–18. doi: 10.1128/mBio.01292-18, PMID: 30108170 PMC6094484

[14] Zhang L Li L Zhang P Cai Y Hua D. LINC00957 Acted as Prognostic Marker Was Associated With Fluorouracil Resistance in Human Colorectal Cancer. Front Oncol (2019) 9:776. doi: 10.3389/fonc.2019.00776, PMID: 31497531 PMC6713158

[15] Wang X Zhao Z Tang N Zhao Y Xu J Li L. Microbial Community Analysis of Saliva and Biopsies in Patients With Oral Lichen Planus. Front Microbiol (2020) 11:629. doi: 10.3389/fmicb.2020.00629, PMID: 32435231 PMC7219021

[16] Vivian J Rao A Nothaft F Ketchum C Armstrong J Novak A. Toil enables reproducible, open source, big biomedical data analyses. Nat Biotechnol (2017) 35(4):314–6. doi: 10.1038/nbt.3772, PMID: 28398314 PMC5546205

[17] Yu G Wang L Han Y He Q. clusterProfiler: an R package for comparing biological themes among gene clusters. Omics J Integr Biol (2012) 16(5):284–7. doi: 10.1089/omi.2011.0118, PMID: 22455463 PMC3339379

[18] Robin X Turck N Hainard A Tiberti N Lisacek F Sanchez J. pROC: an open-source package for R and S+ to analyze and compare ROC curves. BMC Bioinf (2011) 12:77. doi: 10.1186/1471-2105-12-77, PMID: 21414208 PMC3068975

[19] Zhao Y Wang W Liang H Yang C D’Amico T Ng C. The Optimal Treatment for Stage IIIA-N2 Non-Small Cell Lung Cancer: A Network Meta-Analysis. Ann thoracic Surg (2019) 107(6):1866–75. doi: 10.1016/j.athoracsur.2018.11.024, PMID: 30557543

[20] Peng F Wang R Zhang Y Zhao Z Zhou W Chang Z. Differential expression analysis at the individual level reveals a lncRNA prognostic signature for lung adenocarcinoma. Mol Cancer (2017) 16(1):98. doi: 10.1186/s12943-017-0666-z, PMID: 28587642 PMC5461634

[21] Shen Q Jiang Y. LncRNA NNT-AS1 promotes the proliferation, and invasion of lung cancer cells via regulating miR-129-5p expression. Biomed Pharmacother = Biomed Pharmacother (2018) 105:176–81. doi: 10.1016/j.biopha.2018.05.123, PMID: 29857296

[22] Zhai N Xia Y Yin R Liu J Gao F. A negative regulation loop of long noncoding RNA HOTAIR and p53 in non-small-cell lung cancer. Onco Targets Ther (2016) 9:5713–20. doi: 10.2147/ott.s110219, PMID: 27695348 PMC5033503

[23] Li S Mei Z Hu H Zhang X. The lncRNA MALAT1 contributes to non-small cell lung cancer development via modulating miR-124/STAT3 axis. J Cell Physiol (2018) 233(9):6679–88. doi: 10.1002/jcp.26325, PMID: 29215698 PMC13482422

[24] Vendrell J Taviaux S Béganton B Godreuil S Audran P Grand D. Detection of known and novel ALK fusion transcripts in lung cancer patients using next-generation sequencing approaches. Sci Rep (2017) 7(1):12510. doi: 10.1038/s41598-017-12679-8, PMID: 28970558 PMC5624911

[25] Noh J Kim K McClusky W Abdelmohsen K Gorospe M. Cytoplasmic functions of long noncoding RNAs. Wiley Interdiscip Rev RNA (2018) 9(3):e1471. doi: 10.1002/wrna.1471, PMID: 29516680 PMC5963534

[26] Chi Y Wang D Wang J Yu W Yang J. Long Non-Coding RNA in the Pathogenesis of Cancers. Cells (2019) 8(9):1015. doi: 10.3390/cells8091015, PMID: 31480503 PMC6770362

[27] Chen J Liu A Lin Z Wang B Chai X Chen S. Downregulation of the circadian rhythm regulator HLF promotes multiple-organ distant metastases in non-small cell lung cancer through PPAR/NF-κb signaling. Cancer Lett (2020) 482:56–71. doi: 10.1016/j.canlet.2020.04.007, PMID: 32289442

[28] He Y Liu H Wang S Chen Y. Prognostic nomogram predicts overall survival in pulmonary large cell neuroendocrine carcinoma. PLoS One (2019) 14(9):e0223275. doi: 10.1371/journal.pone.0223275, PMID: 31560723 PMC6764685

