# Correction: Upregulation of long noncoding RNA GCC2-AS1 facilitates malignant phenotypes and correlated with unfavorable prognosis for lung adenocarcinoma

**DOI:** 10.3389/fonc.2025.1644707

**Published:** 2025-10-03

**Authors:** Fengqiang Yu, Mingqiang Liang, Weidong Wu, Yu Huang, Jiantao Zheng, Bin Zheng, Chun Chen

**Affiliations:** ^1^ Department of Thoracic Surgery, Fujian Medical University Union Hospital, Fuzhou, China; ^2^ Fujian Provincial Key Laboratory of Cardiothoracic Surgery, Fujian Medical University Union Hospital, Fuzhou, China

**Keywords:** lncRNA, GCC2-AS1, lung adenocarcinoma, The Cancer Genome Atlas, prognosis

There was a mistake in **Figure 3C** as published. **Figure 3C** inadvertently contained incorrect images in the transwell assays of A549 cell lines (left and right) due to an oversight in the processing of a substantial volume of images. The corrected **Figure 3** appears below.

**Figure 3:**
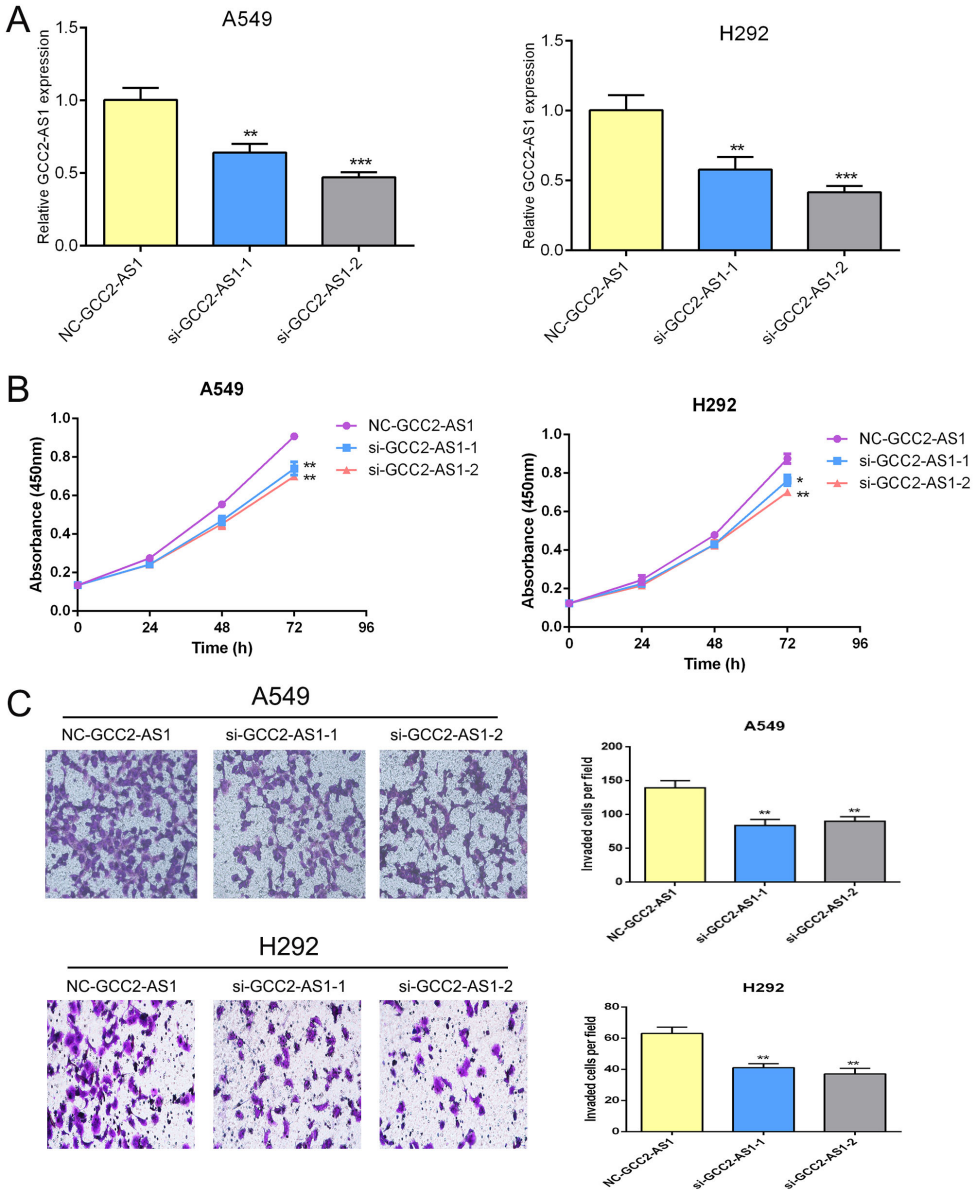
Effects of GCC2-AS1 on lung adenocarcinoma cells. **(A)** Relative expression of GCC2-AS1 in A549 and H292 cells transfected with GCC2-AS1 siRNA and negative control. **(B)** CCK8 assays was performed to detected the proliferation rate. **(C)** Transwell assays were assessed the invasive ability of each group. Scale bar, 20 μm. Data are presented as the mean ± SD of three independent experiments (**P < 0.01; ***P < 0.001).

The original version of this article has been updated.

